# Supplementary material for: Evaluation of miRNA as Biomarkers of Emotional Valence in Pigs
Source: Animals (Basel). 2021 Jul 9;11(7):2054. doi: 10.3390/ani11072054 (PMC8300371; doi:10.3390/ani11072054)
Supplement: Supplementary file 1 [file animals-11-02054-s001.zip › animals-1274899-supplementary.pdf]

Supplementary Materials

Table S1: Indicates the top 10 miRNA genes with highest, albeit non-significant, p-values at bleed 1 in pigs exposed to either enriched or barren housing. Column 1 indicates gene ID; column 2 indicates fold change; column 3 indicates P-value ($p < 0.05$); and column 4 indicates p-values with an adjusted false detection rate (FDR $p < 0.05$).

**Gene ID**	**Fold change**	***p*-value**	**FDR *p*-value**
20525587	6.58	0.0023	0.9513
20533249	-2	0.0034	0.9513
20517267	-3.04	0.0042	0.9513
20524386	-2.2	0.0042	0.9513
20525773	-2.48	0.0067	0.9513
20501671	-2.16	0.0082	0.9513
20525565	3.79	0.0104	0.9513
20514348	-2.72	0.0119	0.9513
20513774	-2.14	0.0143	0.9513
20517052	-2.17	0.0211	0.9513

Table S2: Indicates the top 10 miRNA genes with highest, albeit non-significant, p-values bleed 2 in pigs exposed to either enriched or barren housing. Column 1 indicates gene ID; column 2 indicates fold change; column 3 indicates p-value ($p < 0.05$); and column 4 indicates p-values with an adjusted false detection rate (FDR $p < 0.05$).

**Gene ID**	**Fold change**	***p*-value**	**FDR *p*-value**
20517759	2.14	1.65E-05	0.5116
20519021	-2.88	0.0017	0.963
20525306	-2.9	0.0022	0.963
20504341	-2.36	0.0025	0.963
20508751	-2.36	0.0025	0.963
20509628	-2.36	0.0025	0.963
20516711	-2.36	0.0025	0.963
20524272	2.67	0.0026	0.963
20514111	2.91	0.0031	0.963
20511656	-2.86	0.0034	0.963

Table S3: Indicates the top 10 miRNA genes with highest, albeit non-significant, p-values within the amygdala of pigs exposed to either enriched or barren housing. Column 1 indicates gene ID; column 2 indicates fold change; column 3 indicates p-value ($p < 0.05$); and column 4 indicates p-values with an adjusted false detection rate (FDR $p < 0.05$).

**Gene ID**	**Fold change**	***p*-value**	**FDR *p*-value**
20535149	2.43	7.74E-06	0.2097
20519174	-2.08	4.36E-05	0.2097
20511874	-3.62	4.37E-05	0.2097
20526925	2.11	5.60E-05	0.2097
20529711	-6.27	5.89E-05	0.2097
20519573	2.46	6.36E-05	0.2097
20518793	-4.96	7.52E-05	0.2097
20523375	-4.96	7.52E-05	0.2097
20504842	-5.97	0.0003	0.2711
20501666	-2.55	0.0004	0.277
